# Female Urethroplasty: Outcomes of Different Techniques in a Single Center

**DOI:** 10.3390/jcm10173950

**Published:** 2021-08-31

**Authors:** Marjan Waterloos, Wesley Verla, Michel Wirtz, Mieke Waterschoot, Wietse Claeys, Philippe Francois, Nicolaas Lumen

**Affiliations:** 1 AZ Maria Middelares Gent, 9000 Ghent, Belgium; 2 Ghent University Hospital, 9000 Ghent, Belgium; wesley.verla@uzgent.be (W.V.); michelwirtz5@hotmail.com (M.W.); mieke.waterschoot@uzgent.be (M.W.); wietse.claeys@uzgent.be (W.C.); nicolaas.lumen@uzgent.be (N.L.); 3 Centre Hospitalier de Mouscron, 7700 Mouscron, Belgium; p.francois@chmouscron.be

**Keywords:** female urethral stricture, female urethroplasty, female urethral injury

## Abstract

Introduction: Female urethral strictures and injuries are relatively uncommon compared to males. A wide range of possible causes and treatment modalities have been described. Lately female urethral reconstruction is gaining attention and is fortunately no longer a neglected topic within the reconstructive urology. As such, we aimed to describe our surgical techniques and outcomes for female urethroplasty from a tertiary center. Materials and Methods: Records of female patients who underwent a urethroplasty between July 2018 and May 2021 in our tertiary referral center were reviewed. Patients were subdivided in two groups: patients who suffered from a urethral injury and received an early repair urethroplasty, and patients with a true urethral stricture who received a delayed urethroplasty. Preprocedural, surgical and postoperative data were collected and analyzed with descriptive statistics. Results: A total of five patients in group 1 and nine patients in group 2 were included. Etiology of the urethral injury in group 1 was iatrogenic in 80% and transitional cell carcinoma of the urethra in 20% of cases. A patency rate of 100% at a follow-up of 30 months was achieved with the different techniques. In group 2 etiology was idiopathic (44%), iatrogenic (44%) and due to external trauma in 12% of cases. Urethroplasty technique consisted of primary repair or dorsal onlay of a buccal mucosal graft. Patency rate was 100% at a median follow-up of 13 months. Three patients suffered from postoperative urinary incontinence, one in group 1 and two in group 2. Conclusion: Female urethroplasty is a relatively rare entity within reconstructive urethral surgery. This case series of 14 patients demonstrates that with appropriate surgical techniques, a high patency rate with a low complication rate can be achieved. Further prospective studies with standardized diagnostic workup and follow-up should be performed in order to optimize management strategy.

## 1. Introduction

Female urethral reconstruction can be performed for a large variety of female urethral disorders. Injury of the female urethra and female urethral strictures are relatively uncommon and less known compared to male urethral disease. A wide range of possible causes have been described, and therefore urethral strictures should be considered in all women with refractory Lower Urinary Tract Symptoms (LUTS) [1]. Diagnosis can be challenging due to aspecific presentation and absence of a standardized definition and diagnostic criteria [2].

Management of female urethral strictures can be conservative or surgical. Given the shorter urethral length, there is a potentially greater risk of incontinence and neurosensory dysfunction following female urethral reconstruction [3]. Although historically treatment consisted mainly of endoluminal treatment, in the last few years a trend towards urethroplasty has been observed. According to the recently published European Association of Urology (EAU) guidelines on female strictures, augmentation urethroplasty should be performed whenever endoluminal treatment is no longer a viable option. The use of several flaps (vaginal, labial or vestibular) and grafts (oral mucosa, vaginal or labial) have been reported for urethroplasty [4]. The surgical technique of choice is determined by the surgeon’s experience, stricture characteristics and quality of the local tissue [1,5].

The goal of this study was to determine the different techniques that have been performed in our tertiary referral center and report on the outcomes in this patient population.

## 2. Materials and Methods

Records of female patients who underwent a urethroplasty between July 2018 and May 2021 in our tertiary referral center were retrospectively reviewed.

Etiology of the urethral injury or stricture, results of diagnostic workup, applied reconstructive technique, intraoperative findings, early and late postoperative complications and patency rate were assessed.

**Figure 1:**
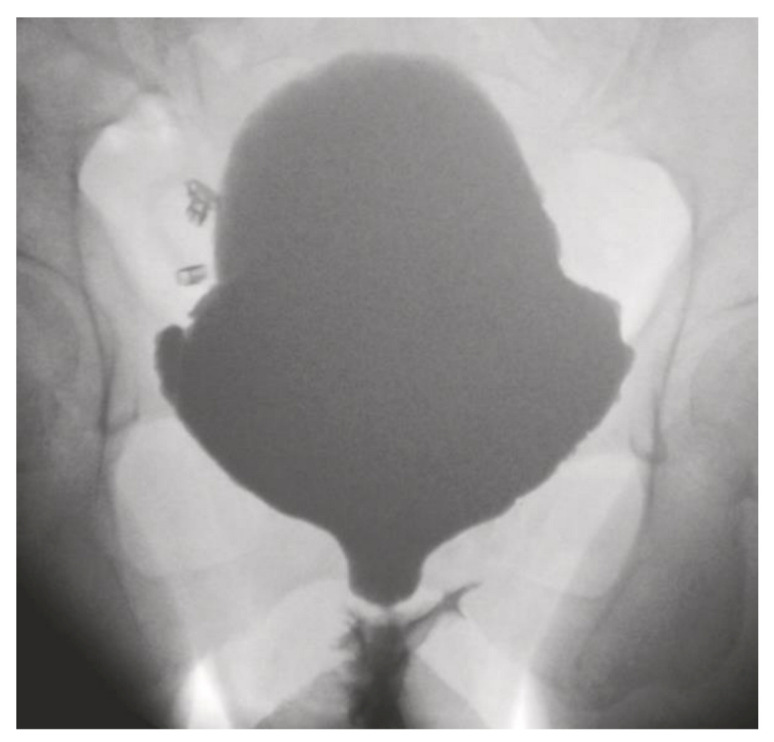
Voiding cystourethrography with a distal female urethral stricture and prestenotic dilation.

Patients who underwent a urethroplasty were subdivided into two groups: one group (1) of patients who suffered from a urethral injury and received immediate reconstruction, and a group (2) of patients who developed a urethral stricture and were treated with a delayed urethroplasty. A urethral stricture was defined as a urethral calibre less than 14 French. All patients with a urethral stricture were evaluated with uroflowmetry, measurement of post void residual volume and voiding cysto-urethrography when feasible (Figure 1).

Postoperatively, patients underwent a voiding cysto-urethrography upon catheter removal and were subsequently followed by uroflowmetry and measurement of the post void residual volume. Patency rate was defined as the absence of lower urinary tract symptoms, a flow rate more than 15 mL/s and normal findings at voiding cysto-urethrography. Descriptive statistics were performed. Informed consents of all patients were registered.

## 3. Results

A total of 14 patients were included, five patients in group 1 and nine patients in group 2. Demographics are available in Table 1.

**Figure 2:**
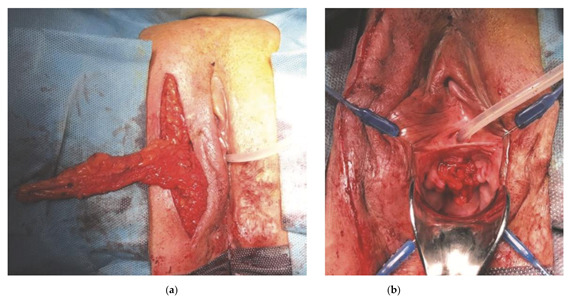
Martius flap interposition (**a**) Prelevation of the Martius flap—(**b**) After transposition of the Martius flap to the ventrally reconstructed area.

In *group 1* (urethral injury) etiology was iatrogenic in four patients caused by urethral injury during surgery for resection of periurethral cysts and intraurethral balloon insufflation. Urethroplasty was performed by debriding the urethral edges, and primary closure in multiple layers over a 16F catheter. One patient with a history of pelvic radiotherapy received additionally a Martius flap interposition to reduce the risk of urethrovaginal fistula formation (Figure 2).

In one patient, urethral injury was caused by resection of transitional cell carcinoma of the urethra, for which an inverted U vaginal flap urethroplasty was performed.

Hospitalisation was short (1–2 days) in most patients. The woman who received a primary repair with Martius flap interposition had a longer hospital stay because of the concomitantly performed Wertheim-Meigs radical hysterectomy.

Catheterisation was needed for 7–21 days, and the catheter could be removed upon the first postoperative consultation after voiding cysto-urethrography. At a median follow-up of 30 months there was a 100% patency rate with at least a maximum flow rate of 15 mL/s. There was no measurable post void residual volume. One patient suffered from mixed urinary incontinence with a strongly reduced bladder capacity caused by previous radiotherapy for a gynaecological malignancy, and was treated medically.

Etiology in *group 2* was idiopathic in four women. In another four patients, iatrogenic injury was caused by erosion of a suburethral Tensionfree Vaginal Tape (TVT) placed for urinary stress incontinence. One patient suffered from a birth related external trauma. All of these patients developed a urethral stricture, for which a delayed urethroplasty was performed. Previous endoluminal treatment (urethrotomy or dilatation) was reported in six out of nine patients. Median follow-up in this group was 13 months.

In the patient group that suffered from suburethral sling erosion, the urethroplasty technique consisted of partial resection of the eroded mesh, debriding of the urethral edges and primary repair of the urethral defect. The patency rate at a follow-up of 16 months was 100%. Two patients had recurrence of urinary stress incontinence after mesh resection and underwent subsequent Burch colposuspension.

Five patients underwent an augmentation urethroplasty with dorsal onlay of a buccal mucosal graft (BMGU).

At a median follow-up of 5 months the patency rate was 100% in all patients. Maximal flow rate on uroflowmetry increased from 6 mL/s prior to surgery to 15 mL/s postoperatively. There was no postoperative urinary incontinence. (Table 2).

## 4. Discussion

Urethral reconstruction in females can be performed for different reasons. There’s a paucity of data covering this topic, and few prospective or standardized studies have been performed.

A urethral injury is mostly diagnosed at the moment of the traumatic impact. A missed urethral injury can lead to development of a urethral stricture. Blunt traumatic injuries, mainly caused by Pelvic Fracture Related Urethral Injury (PFUI) are a rare cause of female urethral injury. Due to anatomical characteristics, women are less prone than men to suffer from urethral injury in the case of a PFUI [6]. Childbirth related urethral or bladder trauma has been reported in 0.5–1% of women during vaginal delivery [7]. Iatrogenic injury caused by insertion of suburethral sling has an incidence of 0.07–2.5%, while mesh erosion in the late postoperative period is observed in 0.03–0.8% of cases [8]. Possible mechanisms are traumatic catheterization, presence of scarring or ischemic tissue or improper surgical technique. Other iatrogenic causes consist of surgical procedures at the urethra or vaginal wall, such as urethral diverticulectomy, resection of peri-urethral cysts or urethral diverticula, transurethral surgery, or irradiation for pelvic malignancies [3].

From a systematic review on published literature of female urethral stricture disease, Sarin and colleagues reported the etiology to be idiopathic in 51.3%, iatrogenic in 32.8%, inflammatory in 9.2% and traumatic in 6.6% of the cases [2]. Multiple diagnostic modalities have been used in the work-up of female urethral strictures. Patient Reported Outcome Measures (PROMs), uroflowmetry, measurement of the post void residual volume (PVR), retrograde urethrography and cysto-urethrography in the case of (nearly) complete obliteration are mandatory for the diagnosis of urethral strictures [1]. Definition of a female urethral stricture is subject to debate and standardization is currently still lacking. Most series report a urethral calibre <14 French as the threshold for a female urethral stricture [1,9].

To our knowledge, no long-term outcome of primary repair after direct injury of the female urethra has been reported. Some authors state a primary repair can be performed if there is no associated infection and reasonable tissue quality. A tension-free anastomosis should be obtained. Whenever there is no sufficient healthy tissue to cover the mucosal defect, a Martius flap interposition should be performed [3]. This was the case in our subgroup of patients who suffered from a direct urethral trauma for which a primary repair was performed with a success rate of 100%. In one patient a Martius flap interposition was needed. The mixed urinary incontinence in one woman was at least partially provoked by previous radiotherapy (radiocystitis).

In our group of patients where a delayed primary repair was performed after mesh erosion and stricture formation, a 100% (four out of four cases) patency rate at a follow-up of 16 months was achieved. Postoperative incontinence was observed in two out of four patients. In this subgroup of patients, removal of the anti-incontinence sling resulted in recurrence of the stress urinary incontinence. This was successfully treated with a Burch colposuspension.

Dorsal onlay BMG urethroplasty has a reported success rate between 62.5–100% at a median follow-up of 6–28 months [10,11,12,13,14,15,16,17,18,19]. These results are corroborated by our series, with a 100% success rate with buccal graft urethroplasty. This group did not show any postoperative urinary incontinence, also in accordance with current literature.

In comparison to the de novo incontinence rate of 0–5.8% as reported in a systematic review by Sarin and colleagues, our de novo incontinence rate of 17.6% (3/17) appears steep. This can mainly be explained by the resection of TVT mesh in two of the three patients. The mixed urinary incontinence in one patient can likely partially be attributed to previous radiotherapy (radiocystitis).

A weakness of this case series is the retrospective analysis of the data. The cohort of patients is small in absolute number; however, it is one of the larger case series in recently published literature.

There might have been under reporting of recurrence rate since calibration or urethroscopy was not routinely performed at follow-up consultation. However, calibration in the absence of symptoms, or a weak uroflow, would only reveal subclinical strictures where there is no indication for treatment.

The absence of standardized questionnaires such as PROMs is also a weak point of this study, as is the absence of a standardized diagnostic and follow-up protocol. We are, however, currently in the process of collecting a prospective database of all urethral reconstructive surgery and are including PROMS as an outcome measure.

Once sufficient follow-up is achieved an update case series can be analyzed.

## 5. Conclusions

Female urethroplasty is a relatively rare entity within reconstructive urethral surgery. The literature consists of a few reviews and small case series. Further prospective studies with standardized diagnostic workup and follow-up should be performed in order to obtain more data and recommendations regarding the diagnosis and management.

## Figures and Tables

**Table 1:** Patient Characteristics.

Patient Characteristics	Subtype	N (%) Urethral Injury	N (%) Urethral Stricture
**Age in years (median)**		35 (27–86)	53 (36–72)
**Number of patients**		5	9
**Etiology**	Iatrogenic (direct trauma)	3 (60)	4 (44)
	Iatrogenic (catheter related)	1 (20)	
	Idiopathic	0	4 (44)
	External trauma (childbirth related)	0	1 (11)
	TCC of the urethra	1 (20)	0
**Risk Factors**	Smoking	2 (40)	0
	Cardiovascular morbidity	1 (20)	1 (11)
	Diabetes	1 (20)	1 (11)
	EBRT	1 (20)	0
**Previous Interventions**	no previous treatment	5 (100)	3 (34)
	1 urethrotomy/dilatation	0	2 (22)
	multiple urethrotomies/dilatations	0	4 (44)
	urethroplasty	0	0
**Preoperative suprapubic catheter**		0	1 (11)
**Follow-up (months)**		31 (17–35)	13 (1–31)

TCC: Transitional Cell Carcinoma. EBRT: External Beam Radiotherapy.

**Table 2:** Results.

**Results group 1 urethral injury**							
**Urethroplasty technique**	**N**	**FU (months)**	**Hospitalisation (days)**	**OR time (minutes)**	**Catheter stay (days)**	**Postoperative incontinence (N)**	**Patency rate (%)**
**Primary repair**	3	35	1	45	12	0	100
**Primary repair with Martius fat pad interposition**	1	30	10	-	21	1	100
**One stage flap urethroplasty (vaginal)**	1	28	2	65	7	0	100
**Overall group 1 (median)**	5	30	1	49	12	1	100
**Results group 2 urethral stricture**							
**Urethroplasty technique**	**N**	**FU (months)**	**Hospitalisation (days)**	**OR time (minutes)**	**Catheter stay (days)**	**Postoperative incontinence (N)**	**Patency rate (%)**
**Primary repair**	4	16	2	89	17	2	100
**One stage graft urethroplasty (dorsal onlay BMGU)**	5	5	2	85	17	0	100
**Overall group 2**	9	13	2	85	17	2	100

FU: Follow-Up; OR: Operation Room; BMGU: Buccal Mucosal Graft Urethroplasty.

## Data Availability

The data presented in this study are available on request from the corresponding author.
